# Large-scale effects of offshore wind farms on seabirds of high conservation concern

**DOI:** 10.1038/s41598-023-31601-z

**Published:** 2023-04-13

**Authors:** Stefan Garthe, Henriette Schwemmer, Verena Peschko, Nele Markones, Sabine Müller, Philipp Schwemmer, Moritz Mercker

**Affiliations:** 1 Research and Technology Centre (FTZ), Kiel University, Hafentörn 1, 25761 Büsum, Germany; 2 Federation of German Avifaunists (DDA), Hafentörn 1, 25761 Büsum, Germany; 3 Bionum GmbH – Consultants in Biostatistics, Finkenwerder Norderdeich 15 A, 21129 Hamburg, Germany; 4 Institute of Applied Mathematics, Heidelberg University, Im Neuenheimer Feld 205, 69120 Heidelberg, Germany

**Keywords:** Zoology, Animal behaviour, Conservation biology, Ecology, Biodiversity, Environmental sciences, Environmental impact

## Abstract

The North Sea is a key area worldwide for the installation of offshore wind farms (OWFs). We analysed data from multiple sources to quantify the effects of OWFs on seabirds from the family Gaviidae (loons) in the German North Sea. The distribution and abundance of loons changed substantially from the period before to the period after OWF construction. Densities of loons were significantly reduced at distances of up to 9–12 km from the OWF footprints. Abundance declined by 94% within the OWF + 1 km zone and by 52% within the OWF + 10 km zone. The observed redistribution was a large-scale effect, with birds aggregating within the study area at large distances from the OWFs. Although renewable energies will be needed to provide a large share of our energy demands in the future, it is necessary to minimize the costs in terms of less-adaptable species, to avoid amplifying the biodiversity crisis.

## Introduction

Accumulating evidence suggests that the Earth’s climate is changing rapidly (e.g. ref.^1,2^), with consequences not only for animals and plants, but also for humans, e.g. in terms of sea-level rise, food security, and health^3–5^. Energy supplies thus need to become more sustainable and secure, in order to reduce greenhouse gas emissions. Generating energy from renewable sources has therefore become a high priority in energy-policy strategies at both national and global levels^6,7^. Wind energy, specifically the installation of offshore wind farms (OWFs^8^), has made a substantial contribution to renewable energy production in recent decades, with the North Sea currently being the key area for OWFs worldwide (www.4coffshore.com^8^).

Top predators, such as marine mammals, seabirds, and large fish, are strongly affected by changes in the environment and lower trophic levels, as well as by anthropogenic activities (e.g. ref.^9,10^). They therefore provide important indicators of the state of our ecosystems for scientific monitoring^11,12^ and for official state of the environment reports, i.e. for the Marine Strategy Framework Directive in European seas (e.g. ref.^13^).

Different seabird species respond differently to the development of OWFs, with behavioural reactions ranging from complete avoidance to attraction^14,15^. A recent review of 20 OWFs in European waters found that species responded differently and sometimes inconsistently across studies, ranging from strong avoidance to strong attraction to OWFs^16^. Avoidance was mainly due to birds responding to OWF structures and was stronger when the turbines were operating, but could also occur as a result of boat traffic to and from the OWFs^17,18^. When avoiding OWFs, species may experience functional habitat loss due to displacement^19^, which could in turn result in increased energy consumption if the alternative foraging habitats are of poorer quality or if the individuals have to travel longer distances to reach their foraging areas^20^. In contrast, if seabirds do not avoid OWFs or are even attracted to them, they risk colliding with the rotors or turbines, leading to increased mortality (reviewed in ref.^21^). However, the possible long‐term effects of the different reactions towards OWFs at the population level are difficult to estimate^20,22^.

Loons (divers, family Gaviidae) belong to the most sensitive group of species with respect to the avoidance of OWFs, as shown by studies of single or small groups of OWF sites in the North Sea, with significant negative effects on loon abundance up to 16 km from the outer edges of OWFs (e.g. ref.^17,23–26^). Furthermore, loons are very sensitive to ship traffic, with long flush distances in front of approaching vessels and significantly lower densities in areas with permanently higher ship traffic^27,28^.

Their sensitive nature and the fact that a significant proportion of their biogeographic population occurs in European waters mean that loons are currently listed in Annex I of the EU Birds Directive, and are rated as particularly threatened by human activities (e.g. ref.^14,15^). One of the most important resting sites for loons is located in the southeastern North Sea, where internationally important numbers aggregate during spring migration^29^ leading to the designation of Special Protection Areas for birds in the Danish and German parts of the North Sea^30^. The main species of loons in these areas are red‐throated loons (*Gavia stellata*; 92%) and, to a lesser extent, black‐throated loons (*Gavia arctica*; 8%^30^).

In this study, we collated and analysed data from multiple sources to quantify the effects of OWFs on loons for the entire German North Sea. Comprehensive monitoring has to be carried out before, during, and after the establishment of OWFs. We aimed to use these long-term and large-scale data sources to describe the large- and medium-scale distributions and abundances of loons before and after establishing OWFs throughout the German North Sea. We also quantified the impacts of 14 OWFs located in five wind farm clusters (i.e. multiple OWF sites in direct vicinity), which became operational between 2010 and 2014, on the small-scale distribution of loons and their numbers in the OWF clusters, including an estimation of disturbance distances. We further discuss the consequences of these impacts on the conservation of loons and other seabirds, and on the management of marine waters in relation to large-scale offshore wind energy utilization. The large scale of this new human activity in marine ecosystems, currently effectively converting 439 km^2^ of marine waters into an industrial area involving 755 turbines producing 3.4 GW of energy (www.4coffshore.com) with associated maintenance ship traffic, can be considered as one of the largest recent human impacts on the marine environment.

## Results

**Figure 1:**
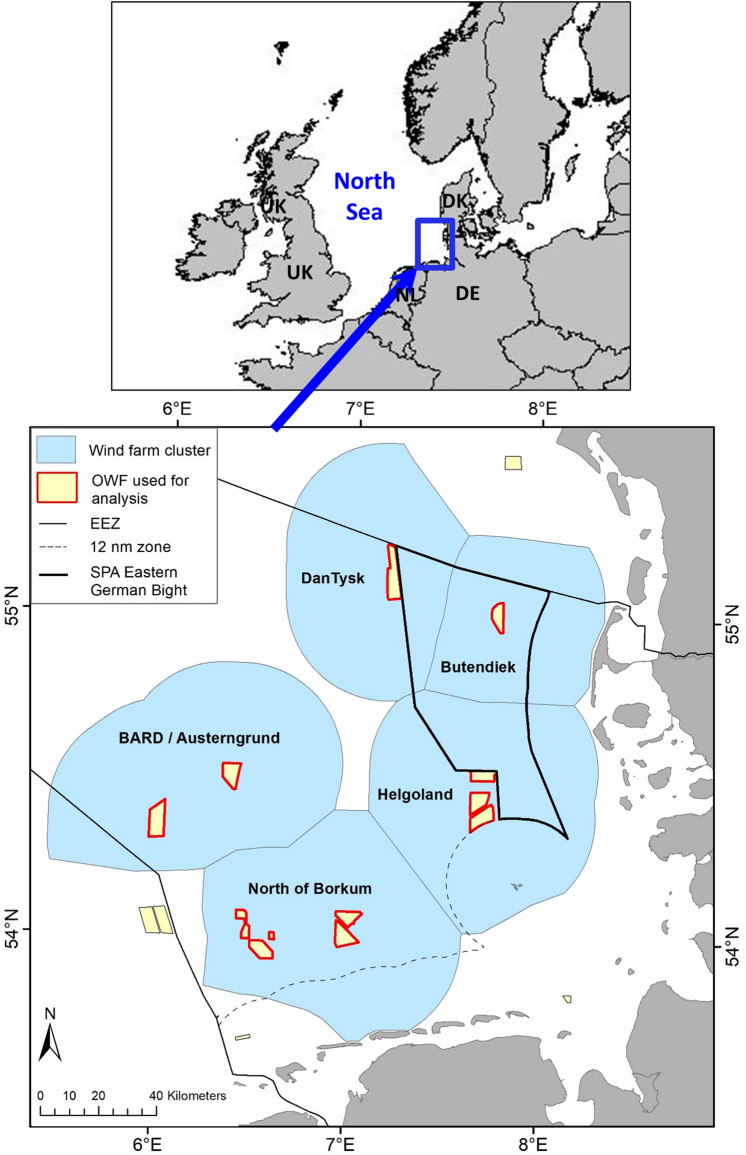
Location of the five wind farm clusters in the southeastern North Sea in northwest Europe. Blue areas delineate the five considered wind farm clusters. The map represents all OWFs in 2015 and 2016. The thick black line indicates the Special Protection Area ‘Eastern German Bight’ designated according to the EU Birds Directive. The thin black line shows the borders of the EEZs (Exclusive Economic Zones) of Denmark (in the north), Germany (in the middle) and the Netherlands (in the south). The map was generated in ArcGIS 10.3 for Desktop, https://www.esri.com.

**Figure 2:**
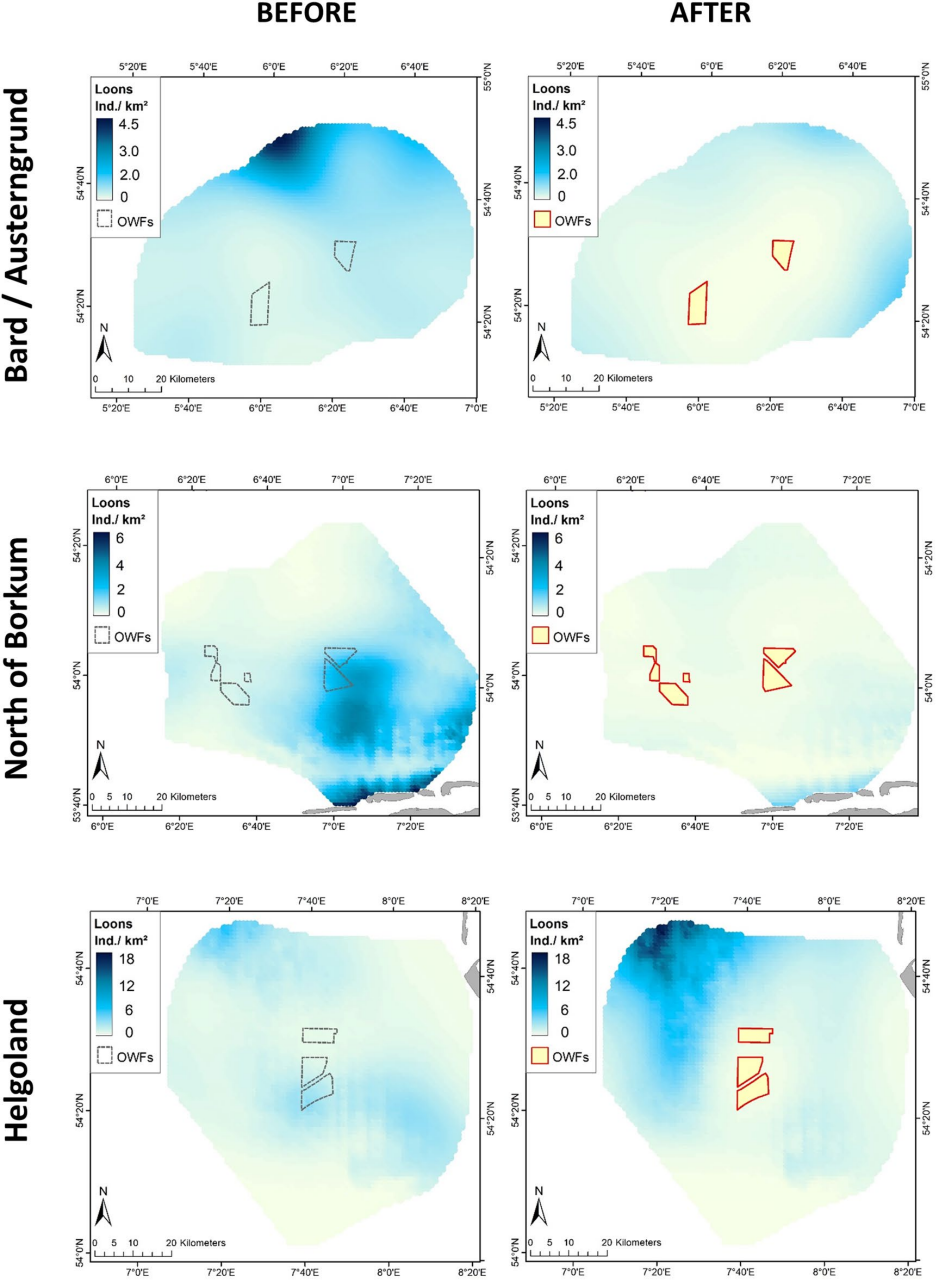

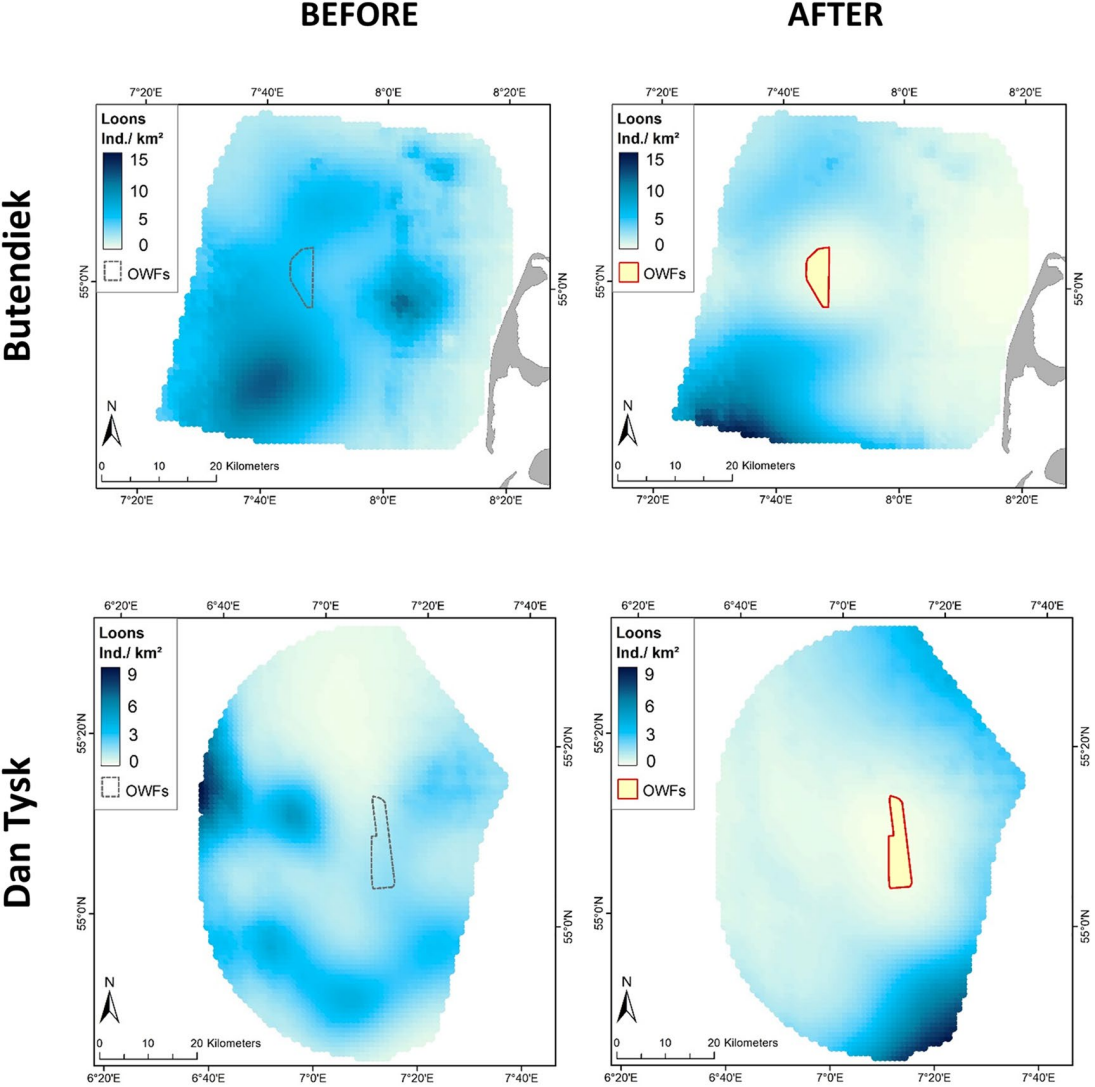
Modelled distributions of loons (individuals per km^2^) before (left panels) and after (right panels) construction of wind farms, shown separately for each wind farm cluster. Note that abundance levels differ between clusters. The map was generated in ArcGIS 10.3 for Desktop, https://www.esri.com.

**Figure 3:**
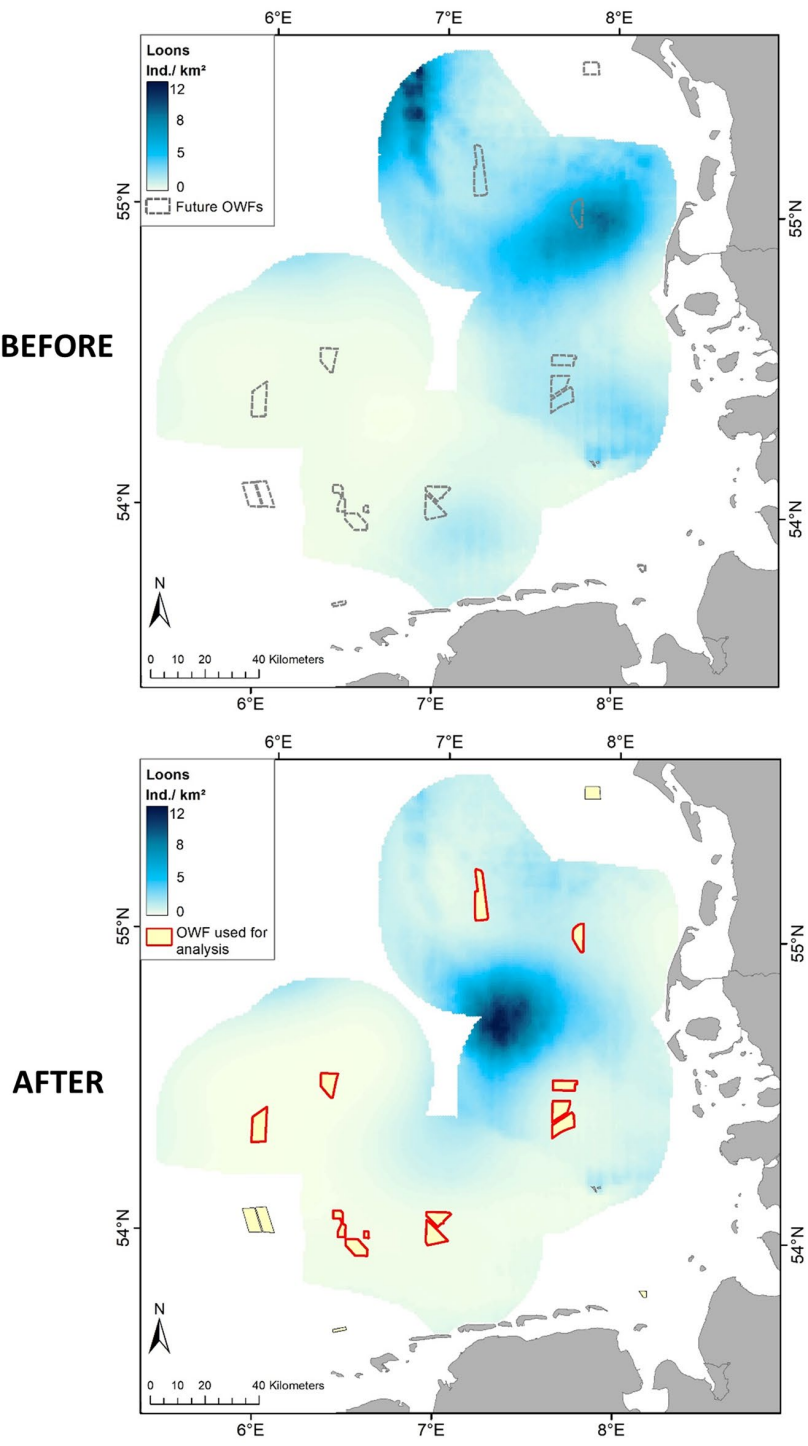
Modelled distribution of loons (individuals per km^2^) before and after construction of wind farms, combined for all five wind farm clusters. Note that abundance levels differ from those in the single clusters, as shown in Fig. 2. The map was generated in ArcGIS 10.3 for Desktop, https://www.esri.com.

The distribution and abundance of loons changed substantially from the ‘before’ to the ‘after’ periods in all five wind farm clusters (Figs. 1, 2). In all cases, the wind farms created a kind of halo around these constructions, with low to very low abundances of loons. Medium to high densities (sky-blue to dark-blue in Fig. 2) were only found at ≥ 10 km from the OWF footprints. In the ‘Helgoland’ cluster, increased aggregation of loons to the northwest of the wind farms appeared after their construction. This coincided with a larger scale re-distribution of birds from the northernmost clusters ‘Butendiek’ and ‘Dan Tysk’, respectively, which exhibited the highest densities of loons before establishment of the wind farms. The most striking differences were the almost complete disappearances of loons in the ‘after period’ in the wind farm clusters ‘BARD/Austerngrund’ and ‘North of Borkum’ (Fig. 2). Re-distribution inside the study area was especially apparent when modelling the combined distribution for all five wind farm clusters. After OWF construction, loons only aggregated in one area, with high densities at large distances from all the existing wind farms (Fig. 3).

**Figure 4:**
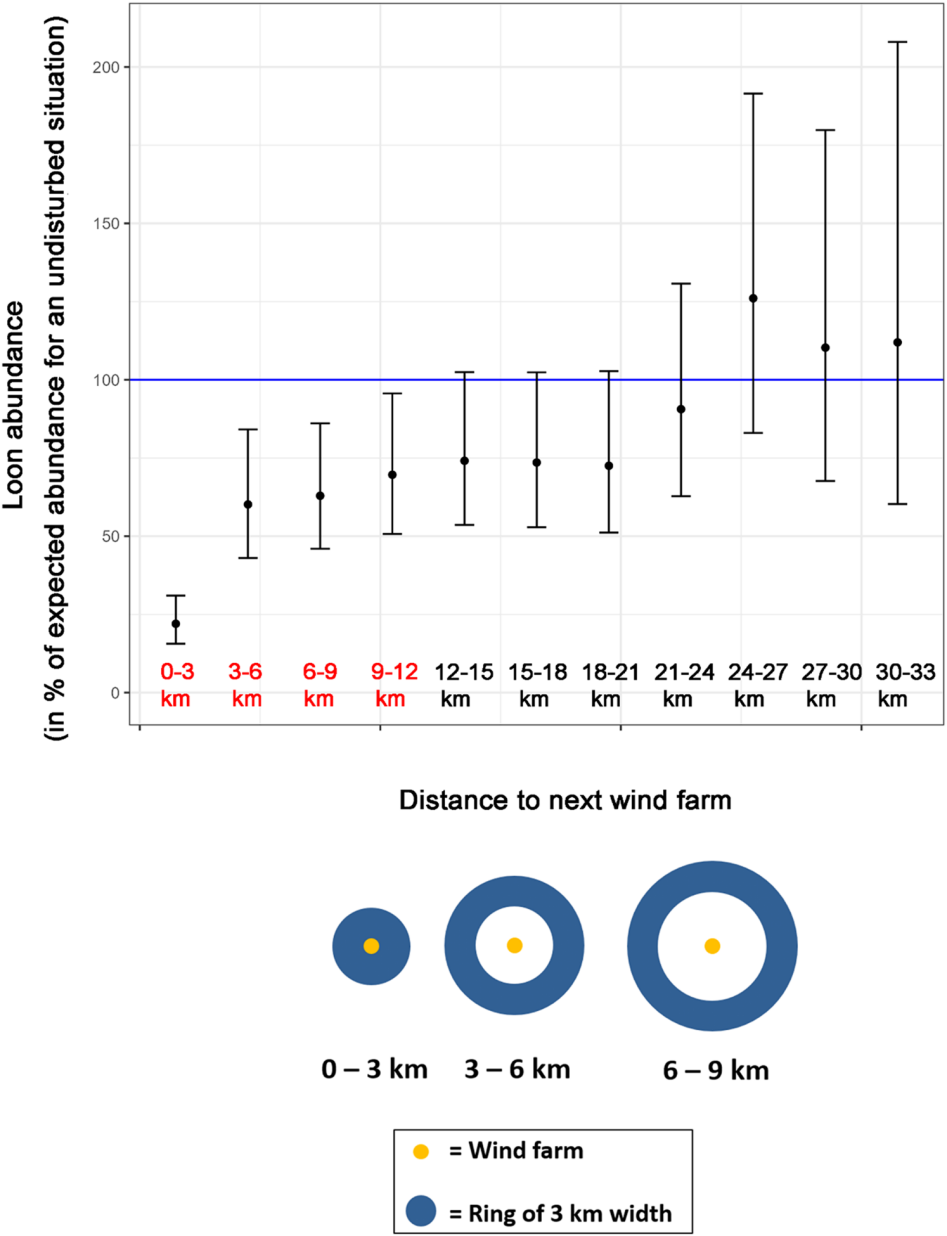
Relative abundances of loons after construction of wind farms in relation to expected abundance without disturbance (100%, blue line). Values shown for steps of 3-km-wide zones (rings) around the wind farms (x-axis); distances in red colour indicate significant reductions of loon abundance. Mean values indicated by dots; 95% confidence intervals indicated by length of bars.

Loon abundance was significantly reduced in the ‘after’ period up to a distance of 9–12 km (mean and confidence interval were well below the expected abundance levels based on the previous ratio during the ‘before’ period, i.e. blue line in Fig. 4). Mean loon abundance was still reduced even at distances up to 24 km, although the effect was not significant.

**Figure 5:**
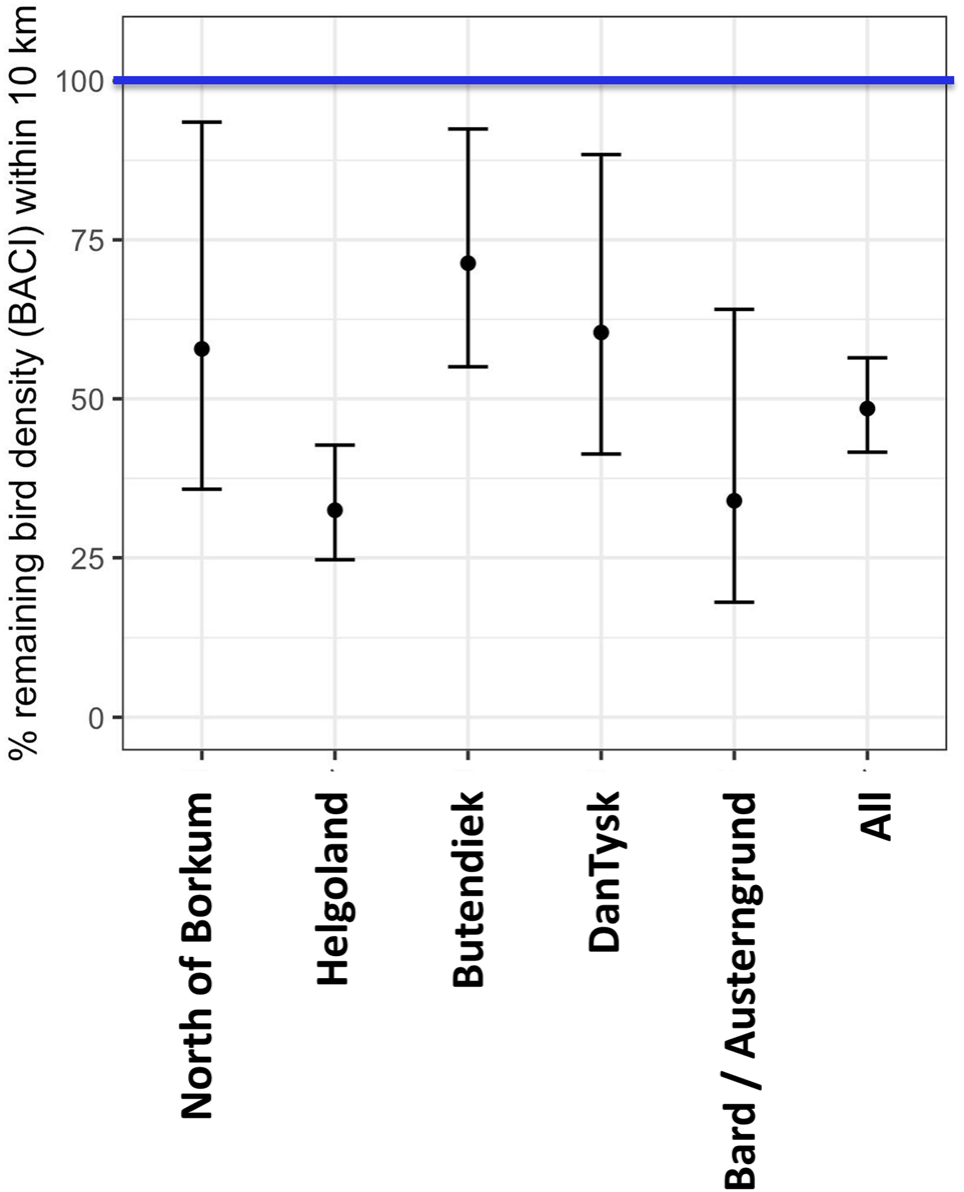
Relative population changes (BACI effect) in the zone within 10 km of OWF compared with the zone outside 10 km of OWF. Data are given for each wind farm cluster as well as for all clusters combined. The blue line represents the abundance of loons in the zone outside 10 km. Mean values indicated by dots; 95% confidence intervals indicated by length of bars.

Loon abundances within the zone of the OWFs + 10 km radius declined strongly from ‘before’ to ‘after’ in all five wind farm clusters, with mean reductions (BACI-effects) of 29–68%, and 52% in all wind farm clusters combined (Table 1, Fig. 5). Abundances fell even stronger for the OWFs + 1 km radius with 94% for all wind farm clusters combined (Table 1). Total population estimates for all wind farm clusters declined by almost a third, from 34,865 individuals before construction to 24,672 individuals after construction. Focusing on the significantly affected 10-km zones around the wind farm clusters, numbers declined from 7750 individuals before to 2893 individuals after OWF construction. This indicated a population decline of 63% within the 10-km zones of all wind farm clusters, and a decline of 20% (27,116 to 21,779 individuals) beyond the 10-km radius.

**Table 1:** Definition of wind farm clusters, years of data analysis, and changes in loon populations determined by BACI analysis.

Cluster	Offshore wind farms	Number of turbines	Installed power (MW)	Area used (km^2^)	Periods (years)		Population change (BACI effect)			
					Before	After	Within 1 km around OWFs		Within 10 km around OWFs	
							Mean	95% CI	Mean	95% CI
Dan Tysk	Dan Tysk	80	288	65.66	2008–2013	2014–2016	− 94%	− 81%, − 98%	− 40%	− 12%, − 59%
Butendiek	Butendiek	80	288	33.12	2009–2014	2015–2017	− 99%	− 94%, − 99.7%	− 29%	− 8%, − 45%
Helgoland	Amrumbank-West, Nordsee-Ost, Meerwind Süd/Ost	208	885.2	108.76	2008–2013	2015–2017	− 92%	− 84%, − 96%	− 68%	− 57%, − 75%
BARD/Austerngrund	Global Tech, BARD 1	160	800	99.35	2005–2010	2014–2016	− 46%	+ 357%, − 92%	− 66%	− 36%, − 82%
North of Borkum	Alpha Ventus, Trianel Windpark Borkum, Borkum Riffgrund 1, Gode Wind 1 and 2	227	1154	132.02	2004–2009	2015–2016	− 94%	− 81%, − 98%	− 42%	− 6%, − 64%
All cluster combined							− 94%	− 91%, − 96%	− 52%	− 44%, − 58%

*BACI* before-after control-impact, *CI* confidence interval.

## Discussion

### Loons and OWFs

This study showed that loons avoided OWFs up to a distance of ca. 10 km and even up to 24 km throughout the German Bight, across all 14 OWFs located in five clusters. The large-scale and long-term data used in this study allowed the first species-specific, comprehensive view of the establishment of OWFs in the marine environment. It confirmed the findings for single OWFs or smaller clusters^17,25^, and revealed a redistribution of loons at a much larger scale (beyond single OWF clusters; see also^26^). Birds aggregated within their original distribution area, but as far away from the OWFs as possible. This strongly suggests that OWFs restrict the spatial freedom of loons, which is likely to be crucial for their ability to react flexibly to prey that is inhomogeneously distributed in time and space^31^. The current stage of OWF development in the German North Sea has clearly led to a reduction of the former loon-distribution area, resulting in a new distribution pattern and a loss of the areas that were preferred prior to OWF construction. This holds true also for the marine protected area “SPA Eastern German Bight” located inside the study area. Loons were among the most important seabird species for designating this area^30^. After the establishment of the OWFs one of the two previous loon hot spots in the study region, located inside the SPA, has disappeared and the only one new distribution hot spot is partly located outside the SPA. Consequences of these changes for the functioning of the SPA concept need to be investigated. Large areas in the south and southwest of the study area were largely abandoned after the establishment of five OWFs and their associated OWF supply- and maintenance-vessel traffic. This change was significantly related to the avoidance of the proximity of wind farms. Densities in other areas have increased, potentially leading to increased competition for already less valuable habitat, with energetic consequences. Data on foraging conditions are currently lacking, and this would be difficult to assess due to the temporal and spatial variability of their prey. However, population regulation theory, as well as observational evidence, suggest that birds generally occupy higher quality habitat first, in preference to poorer habitat^32^. Red-throated loons are generalist, opportunistic feeders that mainly prey on bentho‐pelagic schooling fish in the German North Sea, including clupeids, mackerel, flatfish, gadoids, and sandeels^33^. They are generally able to switch their prey spectrum to account for seasonal variations in availability, as shown for a herring spawning area in the Baltic Sea ^34^. Loons occur in a regularly productive transition zone between lower saline waters near the coast and more-saline waters offshore (ref.^35^; for a hydrographic description see ref.^36^). However, recent telemetry data from our study area showed that some individuals preferred more coastal waters with lower salinity, while others preferred offshore areas with higher salinity, potentially reflecting individual foraging-site fidelity^25^. When foraging for their highly mobile prey, loons are now more restricted in terms of their larger-scale movements because their former foraging area has been split into smaller units by the establishment of several OWFs.

The estimated abundances of loons in the wind farm clusters before and after construction also showed major changes, with a decline of 63% within the significantly affected 10-km zone around the wind farms compared with only a 20% decline beyond 10 km from the OWFs. The decline in numbers beyond 10 km might be due to higher-order effects on loon populations or to local fluctuations in prey populations^37^, but might also mirror cumulative responses of loons to generally increased human pressure associated with ship traffic^17^ and other uses (for an overview of uses see ref.^38^). A correction of the 63% decline in the 10-km radius (corresponding to a multiplicative factor of 0.37) with the 20% decline outside this radius (a multiplicative factor of 0.80) leads to a corrected factor of 0.37/0.80 = 0.46, thus a decline of 54% that might be solely attributable to the construction of the OWFs. Although the ‘before’ and ‘after’ time periods varied among the different wind farm clusters, our data clearly showed consistent and strong patterns.

Population consequences remain unclear to date, and in the absence of empirical evidence, it is difficult to draw concrete conclusions about how displacement will affect individuals and populations^39^. Nevertheless, reduced availability of prime habitat and the consequently inferior foraging options may represent risks for loon populations through reduced body condition, delayed departure to breeding areas, and lower reproductive success, with negative effects on population trends^39^. The observed decline in numbers from before to after the establishment of the OWFs indicates a strong negative pressure on loons in the southeastern North Sea.

### Indirect effects of OWFs on loons

The construction of OWFs also affects the underwater habitats, especially when hard substrates are introduced into an area of mostly sandy sediments^40^. Fish and marine mammals might then be attracted to the vicinity of operating OWFs due to the ‘artificial reef’ effect^41,42^, and an increased diversity and abundance of fish (e.g. ref.^43^) make these areas potentially valuable feeding grounds, as shown for harbour seals^44^. Furthermore, OWFs may function as no fishing/restricted activity zones and thus support fish populations^45^. However, despite the potential surplus of prey within OWF sites, no habituation to the presence of OWFs has yet been recorded for loons, based on data for up to 3 years after establishment of the respective OWFs. It thus remains questionable if the higher fish abundance inside and close to OWFs will support loons, because of the large distances that they maintain from the OWFs.

## Conclusions and perspectives

The current study demonstrated the clear benefit of simultaneously analysing the effects of multiple OWFs, thus enabling generalizations that cannot be made based on single-site assessments. This approach revealed a clear and consistent pattern in which loons avoided OWFs, with significantly reduced abundances up to and exceeding distances of 10 km. No other seabird species group has demonstrated such a strong, negative response to OWFs (cf.^16^), although previous studies also showed significant impacts for other species. In this context it might be noteworthy to refer to recent studies of seabirds breeding on Helgoland, the only seabird cliff in the southern North Sea, located in the center of our loon study area. GPS-tagged common guillemots (*Uria aalge*) from Helgoland avoided OWFs during the operational stage^18^. Similarly, northern gannets (*Morus bassanus*) predominantly avoided OWFs in different years, but a few frequently entered them when foraging or commuting between the colony and foraging areas^46^. OWFs also had strong effects on black-legged kittiwakes (*Rissa tridactyla*), with a similar approach to the current study showing decreases of 45% in the OWF area in the breeding season and 10% in spring, compared with numbers before construction^47^.

Compared with the above studies, loons appear to be more severely affected by the implementation of renewable energy structures at sea. More OWFs in the German sector of the North Sea, as well as in other areas of the North Sea, may lead to further re-distributions and population reductions of these species, but this can only be evaluated by similar investigations in other countries. We recommend increasing consideration and study of cumulative effects of wind farms, throughout the year, and across all relevant regions. This will require greater cross-border collaboration between research institutions, governments, and developers. Nevertheless, the current results suggest that further large-scale habitat restrictions are likely to severely impact this species group.

The fact that several other seabird species exhibit some kind of avoidance behaviour towards OWFs (see studies from Helgoland above) shows that loons are not a single problematic group of birds. Scientifically sound analyses of a variety of species at different locations should help to reveal which marine waters are best-suited for establishing OWFs, without sacrificing species-conservation targets. We have no doubt that renewable energies should provide a large share of our future energy demands, but decisions taken at political and societal levels should also take into account the existing biodiversity crisis^48^ and thus aim to minimize the costs to less-adaptable species for which the North Sea has long provided a predictable food source. These factors are especially relevant in the North Sea, as one of the most‐intensively used sea areas worldwide in terms of human activities such as fishing, transport, oil and gas drilling, and gravel extraction^38,49,50^.

## Methods

### Study concept

We analysed available data for the whole of the German North Sea during the spring periods from 2000 to 2017 from environmental impact studies, monitoring during the construction and operation of wind farms, the German Biodiversity Monitoring project, and from research projects. Data from these different sources and from different counting platforms (ship, aircraft, digital aerial surveys) were harmonized and combined for joint analysis to quantify the effects of OWFs on loons over the entire German North Sea.

### Experimental design

We implemented a ‘before-after control-impact’ (BACI) approach (comparing an ‘impact area’ and a ‘control area’ monitored before and after activation of the impact; ref.^51,52^).

BACI studies can be evaluated using regression techniques^51^, allowing count data to be modelled with appropriate probability distributions in the framework of generalised linear modelling^53,54^, and highly nonlinear dependencies (as frequently observed in ecological systems) to be described based on additive modelling^55,56^.

In the present study, we employed a combination of the above modelling approaches to obtain unbiased results, because we were dealing with both overdispersed count data and highly nonlinear relationships (such as dependency of the abundance on spatial coordinates). This led to a BACI analysis formulated within the framework of generalised additive models (GAMs), as an analytical method that is well-established in the context of ecological studies and has frequently been used in statistical ecology studies (e.g. ref.^54,56,57^).

### Raw bird-count data

Bird-count data were obtained from the above data sources. The data were collected using three different counting platforms (ship, aircraft, digital aerial surveys) and were harmonised^58^ and combined for joint analysis. The study was restricted to the most important period for red-throated loons in the southeastern North Sea (spring staging, 1 March – 30 April; see^29^ for a description of the phenology of this species group in the southeastern North Sea).

The data comprised 25,077 observations of loons and a total survey effort of 47,985 km^2^. The data were assigned to five wind farm clusters based on the minimum distance to the next OWF (after construction) (see Fig. 1 and below).

### Correcting for incomplete bird detection

Bird detection is known to be incomplete, at least for observer-based surveys (aerial or ship-based). On one hand, the probability of detection may decrease with increasing distance from the observer, with the shape and strength of the distance-dependent decrease also depending on other covariates, such as survey method, bird flock size, or sea state^59^. On the other hand, the detection probability on the transect line (i.e. distance-independent detection) may also vary among methods and may depend on additional covariates, such as sea state. To account for the first issue, we applied distance-sampling methods^59^ to the observer-based raw data, where model selection (with respect to detection functions as well as the predictors sea state, bird flock size, log(bird flock size), and their interactions) was applied separately for aerial and ship-based data. The best detection function was subsequently used to correct the case sensitive raw-data (i.e. depending on distance class, method, and all other covariates appearing as predictors in the best detection model). Raw data from digital-based aerial surveys were not distance-corrected, assuming that the detection probability was principally independent of distance^60,61^.

To account for differences in detection on the transect line, the detection-related variables *sea_state* and *method* were used as predictors in the final regression analysis (i.e. applied to distance-corrected data), such that relative differences between methods were considered and estimated. Indeed, all three methods have overlapping time frames, and it was therefore possible to estimate the differences by including the survey method as a covariate in the model.

More technical details on distance-dependent and distance-independent corrections of incomplete bird detection are given in^61^.

### Data pooling

To achieve a manageable degree of spatial autocorrelation, it was necessary to pool the (distance-corrected) raw data. Data were spatially pooled in a grid with cells of 1 × 1 km, separately for each method (observer-based aerial survey vs. observer based ship survey vs. digital aerial surveys) and period (before construction vs. after construction). This grid size appeared to be large enough to produce moderately autocorrelated data, but small enough to provide sufficient spatial resolution. During spatial pooling, bird numbers and the monitored area were summed for each grid cell, whereas geographical coordinates and environmental covariates (see below) were averaged.

### BACI-model covariates

In addition to the detection-related categorical covariates *sea_state* and *method*, we also considered smooth terms for the location-specific variables *dist_land* (nearest distance to the mainland) and *depth* (mean water depth). The aim was to further reduce the amount of unexplained variance and thus increase the power and quality of the predictions in the final regression models. The variables *dist_land* and *depth* were given in an extra data sheet on a regular grid with a high spatial resolution of 0.2 × 0.2 km and were transferred to the pooled bird-count data using cubic interpolation. *Dist_land* was calculated in ArcGIS 10.3 and *depth* data were provided by the Federal Maritime and Hydrographic Agency (BSH).

We introduced a 2D-spatial smooth predictor depending on longitude and latitude to account for additional spatial-abundance heterogeneities not explained by the other used covariates. Because 2D thin plate splines used as spatial smooth predictors are optimised for variables on the same scale^56^, we rescaled the geographical coordinates to kilometres before analysis. For all smooth terms, the optimal degree of smoothing was determined based on generalised cross-validation methods^56^. Neither the spatial smooth nor the above environmental covariates were allowed to vary between the periods (before vs. after construction). They therefore accounted for long-term spatially inhomogeneous bird densities, but did not interfere with the introduced variables for investigating changes in patterns and densities due to wind farms.

To investigate the influence of wind farm presence on bird distribution patterns and abundance, 'before construction' and 'after construction' periods were defined for each wind farm cluster separately, and introduced as an additional binary variable period. The 'before-construction' period was defined as the period before any construction work took place (Table 1), and the 'after-construction' period included the period of construction activities above sea level, because preliminary analyses showed the same type of responses by loons during this phase and the operational phase. To minimize the effect of possible long-term changes in loon abundance and distribution, the maximum duration of each period was restricted to 6 years (Table 1).

The distance to the edge of the nearest OWF was given by the variable *dist_owf*. In the before-period, this variable measured the distance to an OWF that did not yet exist, in order to evaluate changes in response to this variable in the before- vs. the after-period. All data for up to 35 km from any wind farm were used. Larger distances were not expected to disturb the birds though the home range sizes of satellite-tracked birds^62^ suggest that they would be able to commute over such distances.

If the change in abundance within a distinct impact area (delimited from control area) was investigated, the binary variable *B_dist_owf* was defined (based on *dist_owf*), e.g. defining a certain proximity to a wind turbine as 'inside OWF' or 'outside OWF'. *B_dist_owf* was used in regression analyses as a main effect and in interaction with the variable period. The interaction term *B_dist_owf*:*period* represents the relative change in abundance in the impact vs. control areas due to the OWFs. It measures the relative difference in abundances in the impact vs. control areas in the after-period, and corrects this value for the 'natural difference' between these areas in the before-period. The estimated regression coefficient from this interaction term is called the BACI value.

To investigate the disturbance distance, we defined an impact area represented by a 'ring' or 'belt' around the OWFs, with an inner distance radius x and outer distance radius y to the next OWF (Fig. 4, bottom). The control area was given as the area outside this belt and the area inside, not covered by the belt, was excluded from the regression. We then increased the diameter of the belt (using an annulus width of y − x = 3 km for each OWF) and repeated the BACI analysis. The width of 3 km was chosen to allow for a precise spatial presentation of the effects while maintaining a sufficiently large database. This was increased stepwise to determine the distance up to which the BACI effect within the belt was still significant, corresponding to an estimate of the disturbance distance.

To investigate the overall effect of OWFs on loon abundance, we defined *B_dist_owf* such that > 10 km from an OWF was assigned as outside OWF and shorter distances as inside OWF. This order of magnitude was based on previous studies^17,25^ that showed strongly reduced loon abundance within a distance of 10 km around OWFs.

To investigate the distribution patterns and census estimates in the before- vs. after-periods, *dist_owf*-related variables were excluded from the regression models. The dependency on *depth*, *dist_coast*, and the spatial smooth were allowed to vary with the period, and period was introduced only as a main effect. The distribution patterns and bird numbers were thus estimated separately for both periods, completely independent of any information on OWF location, leading to objective estimates of the abundance and distribution patterns.

### BACI regression model structure

We evaluated the BACI data in the framework of GAMs, which allowed us to adapt the analysis to various characteristics of our data, such as overdispersed count data (requiring generalised modelling; ref.^54,63,64^), offset-modelling (because counts have to be related to varying sizes of monitored areas; ref.^63,64^), and strongly nonlinear dependencies (requiring additive modelling; ref.^54,56,57^).

The most complex BACI-GAM (not yet thinned regarding its predictors, as described in the following subsection) is given by:

1 $$\begin{aligned} {\text{log}}\left( {{\text{y}}_{{\text{j}}} } \right) & = \,\beta + {\text{ method}}_{{\text{j}}} + {\text{ sea}}\_{\text{state}}_{{\text{j}}} \\ & \quad + {\text{s}}\left( {{\text{depth}}_{{\text{j}}} } \right) \, + {\text{ s}}\left( {{\text{dist}}\_{\text{coast}}_{{\text{j}}} } \right) \\ & \quad + {\text{s}}\left( {{\text{latitude}}_{{\text{j}}} ,{\text{ longitude}}_{{\text{j}}} } \right) \\ & \quad + {\text{ B}}\_{\text{dist}}\_{\text{owp}}_{{\text{j}}} + {\text{ period}}_{{\text{j}}} \\ & \quad + {\text{B}}\_{\text{dist}}\_{\text{owp}}_{{\text{j}}} :{\text{period}}_{{\text{j}}} \\ & \quad + {\text{offset}}\left( {{\text{log}}\left( {{\text{area}}_{{\text{j}}} } \right)} \right) \, + e_{{\text{j}}} ,\,\, \end{aligned}$$

where *e*_j_ $\upepsilon$ N(0, s2) is independent and identically distributed. Here, y_j_ is the vector of bird numbers, where the index *j* refers to the row of data. $\beta$ is the intercept, s(.) depicts a cubic respectively thin plate regression spline, where the optimal number of knots has been estimated by generalised cross-validation. For each fitted model, an appropriate probability distribution and appropriate subset of predictors were selected based on Akaike information criterion (AIC) analysis^65^ (c.f., following subsection). Importantly, the interaction term B_dist_owp_j_:period represents the relative effect of the wind farms on bird abundance (i.e. within the impact area), corrected for natural/externally driven variations in abundance (i.e. outside the impact area) between the two different periods^51,52^.

Distribution pattern and census were analysed using a slightly modified version of model (1):

2 $$\begin{aligned} {\text{log}}\left( {{\text{y}}_{{\text{j}}} } \right) & = \beta + {\text{ method}}_{{\text{j}}} + {\text{ sea}}\_{\text{state}}_{{\text{j}}} \\ & \quad + {\text{period}}_{{\text{j}}} + {\text{ s}}\left( {{\text{depth}}_{{\text{j}}} ,{\text{ by }} = {\text{ period}}} \right) \\ & \quad + {\text{s}}\left( {{\text{dist}}\_{\text{coast}}_{{\text{j}}} ,{\text{ by }} = {\text{ period}}} \right) \\ & \quad + {\text{s}}\left( {{\text{latitude}}_{{\text{j}}} ,{\text{ longitude}}_{{\text{j}}} ,{\text{ by }} = {\text{ period}}} \right) \\ & \quad + {\text{offset}}\left( {{\text{log}}\left( {{\text{area}}_{{\text{j}}} } \right)} \right) \, + e_{{\text{j}}} , \end{aligned}$$

where the term 'by = period' indicates that smooth terms were estimated independently for each period.

### Model validation strategy

To obtain and validate optimal GAM models, we modified the selection and validation strategies as described e.g. by ref.^63,64,66^). This included carrying out the following steps separately for each model:

1. Based on the “maximal complex model” given above, choose an appropriate probability distribution^65^. Namely we compared Poisson-, negative binomial-, Tweedie-, and zero-inflated Poisson-distributions.
2. Using the most appropriate probability distribution, select an optimal subset of predictors (based on lowest AIC value). We permuted over all possible combinations and formulations of dispensable predictors, and compared 16 different models.
3. The model with the favoured probability distribution and subset of predictors was validated (mainly relying on graphical analysis via residual plots; 66) to test all the required model assumptions, including spatial autocorrelation.

### Calculation of distribution maps and total bird numbers

We calculated distribution maps and total numbers of loons in the before- vs. after-periods using the model based on Eq. (2). We used the fitted model to predict bird densities on a prediction map of the investigated area with a resolution of 1 km^2^, including values for different environmental covariates.

Regarding the detection-related variables *method* and *sea_state*, we investigated which *method*–*sea_state* combination led to the best predictions (i.e. highest detections on the transect line), and subsequently used this combination in the prediction routine. This assumes that at least for one *method*–*sea_state* combination, the detected bird numbers (after distance correction) were close to the real bird numbers, i.e. detection was close to 100%.

### Statistical analysis

All statistical analyses, validation procedures, and visualizations were carried out using the statistical software R^67^. We also used the following packages: *ggplot2*^68^ for visualizations and plots, *MASS*^69^, *pscl*^53^, and *mgcv*^56^ for regression analyses, and *Distance*^70^ for distance sampling-related procedures.

## Data Availability

Data are stored at the German Federal Maritime and Hydrographic Agency (BSH, Hamburg, Germany, https://www.geoseaportal.de/mapapps), and the German Federal Agency for Nature Conservation (BfN, Bonn, Germany, https://geodienste.bfn.de/seevogelmonitoring), and available on request.
